# Effects of physical therapy on quality of life in osteoporosis patients - a randomized clinical trial

**DOI:** 10.1186/1477-7525-10-101

**Published:** 2012-08-24

**Authors:** Guido Schröder, Andreas Knauerhase, Guenther Kundt, Hans-Christof Schober

**Affiliations:** 1 Klinikum Südstadt Rostock, Department Internal Med, Südring 81, Rostock, 18059, Germany; 2 Department Internal Med, University of Rostock, Germany, E.-Heydemann-Str. 6, Rostock, 18057, Germany; 3 Institute for Biostatistics and Informatics in Medicine and Ageing Research, University of Rostock, E.-Heydemann-Str., Rostock, 818057, Germany

**Keywords:** Osteoporosis, Physical therapy, Sling exercise therapy, Quality of life

## Abstract

**Summary:**

The aim of this prospective randomized single-center study was to investigate whether sling exercise therapy is superior to conventional exercises in osteoporosis patients.

**Background:**

Patients with osteoporosis frequently experience fractures of the vertebral body, which may cause chronic back pain and other symptoms. These, in turn, may lead to immobilization, muscular atrophy, and restrictions in activities of daily living. The situation can be improved with specific medication and physiotherapy. We explored the effects of a variety of physical treatments on activities of daily living in patients with osteoporosis.

**Method:**

Fifty patients were randomly allocated to two treatment groups. Group A received traditional physiotherapy (PT) while group B underwent sling exercise therapy (ST). Both treatments were given twice a week for three months. The results of the treatment were registered on the quality of life questionnaire (Qualeffo-41) devised by the International Osteoporosis Foundation. After a further three months with no specific exercise treatment, we again tested all patients in order to draw conclusions about the long-term effects of both types of exercise.

**Results:**

Forty-four patients (88%) completed the study. Patients were assigned to small groups (a maximum of 5 patients in each group) and thus received individual attention and motivation. Quality of life was improved in both groups; a significantly greater improvement was registered in patients who performed sling exercises (Global score Qualeffo: p = 0.002).

**Conclusion:**

The test results confirm the known positive effects of physical therapy on the quality of life of osteoporosis patients, as well as the fact that sling exercises are a sound alternative treatment for this condition.

## Background

Osteoporosis is one of the ten most common illnesses [1]. Approximately 30% of women and 10% of men older than 50 years of age suffer from the disease [2]. Osteoporosis a systemic skeletal disease in which bone mass is reduced, the microstructure of bone destroyed, and the risk of fracture increased [3]. As no early symptoms other than non-specific back pain are observed, osteoporosis is rarely diagnosed before the first fracture occurs [4]. Vertebral fractures are followed by chronic malaise and are known to restrict the mobility of the trunk and lower extremities. This, in turn, increases the risk of falls [5,6]. Fractures are frequently followed by isolation and restriction of independence, leading to loss of autonomy and diminished quality of life in advanced age [7].

A variety of interventions have been developed to address this condition. The benefits of regular physical exercise include reduction of pain, prevention of falls, and improvement of mobility and quality of life [8-13]. Fitness factors such as strength, stamina, and easy movement are encouraged. Owing to the geriatric aspect of osteoporosis, activation of the sensomotor system is important. Many elderly individuals suffer from comorbidities (such as deterioration of visual acuity and perception of contrast) which, however, culminate in physical imbalance only when combined with proprioception deficits.

As recent advancements in sports medicine have proven the beneficial effects of sensomotor exercise on the regulation of posture and/or the frequency of falls [14,15], we decided to use sling exercise therapy. The procedure was developed by Kirkesola [16] to treat motor problems, and was established by Meier [17] for prevention as well as rehabilitation of professional German sportsmen.

To our knowledge, the question as to whether sling exercise improves quality of life in osteoporosis patients has not been previously investigated.

## Method

### Design and randomization

Patients were recruited from the osteoporosis outpatient clinics of Klinikum Südstadt hospital and the University hospital of Rostock. All study participants had proven osteoporosis (T-score ≤ −2.5). Patients who were unable to complete the entire training program during the period of the investigation were excluded from analysis.

Patients were randomized on a 1:1 basis. The permuted block design was used for randomization [18]. The block size was randomly selected. The randomization envelopes were numbered in ascending order. A proband to be randomized opened the envelope with the lowest number among all sealed envelopes.

### Intervention

**Figure 1:**
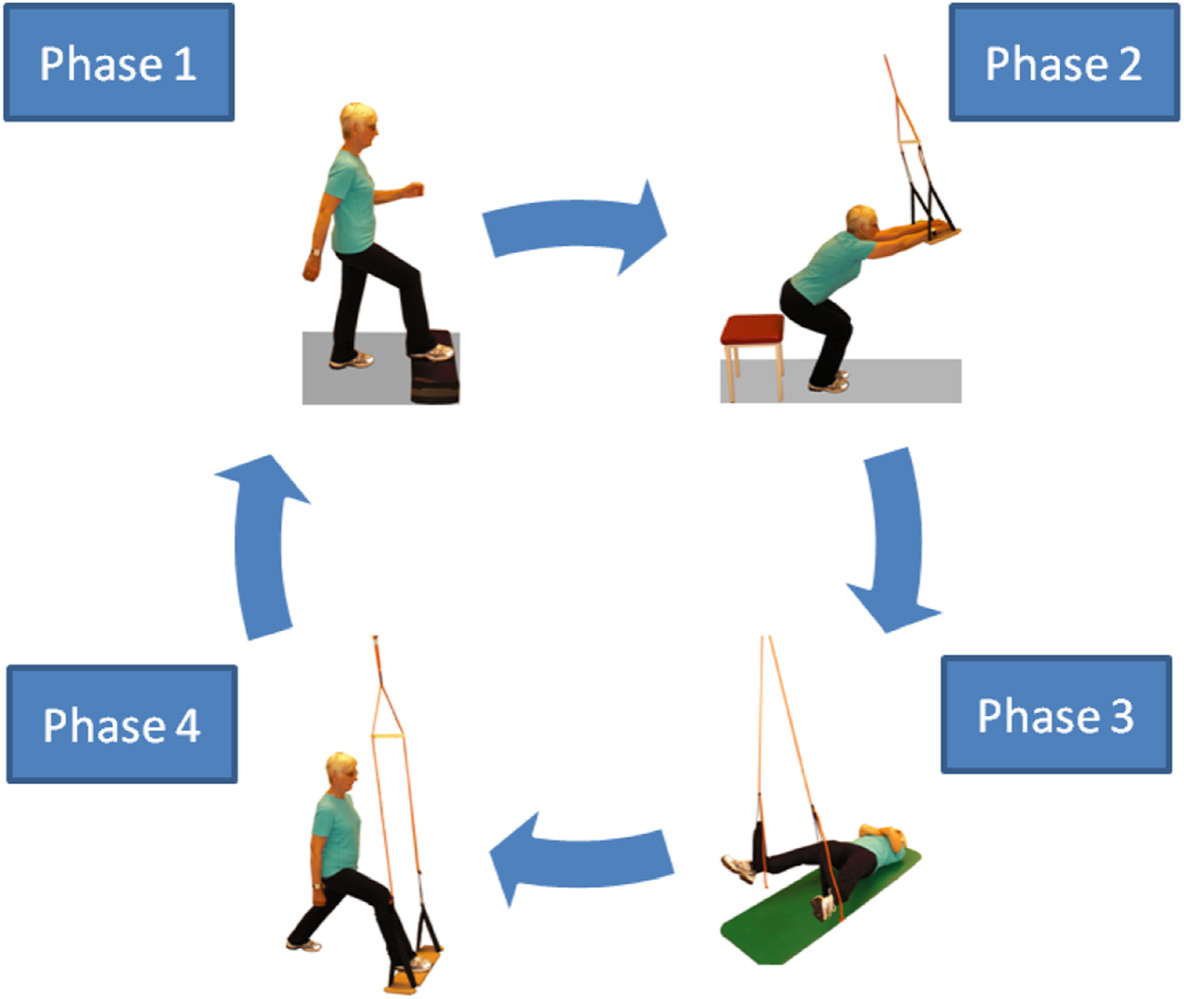
Sling training.

Over a period of 3 months, 44 patients with osteoporosis completed a twice-weekly 30-minute intensive exercise program designed to stabilize the trunk. Both groups completed a training program that consisted of 5 phases. Exemplified in Figure 1 are the 4 main phases of the Sling training represented. Phase 2 had in the course of the intervention no longer be practiced explicitly. The PT group completed similar exercises without slings (eg chair-rising exercises pelvic lift, step-ups).

Phase 1: Systematic cardiovascular and neuromuscular warm-up (PT group: general keep-fit exercises; ST group: step aerobics)

Phase 2: PT and ST groups: functional strength exercises focusing on correct posture

Phase 3: PT and ST groups: functional strength exercises for global surface muscles of the torso; ST group: dynamic sling exercise

Phase 4: Segmental stabilization (SST), both static and dynamic (PT group: exercise/medical ball; ST group: sling)

Phase 5: Stretching and relaxation

Each exercise session included all of the five phases.

### Measurement of quality of life using the Qualeffo-41 questionnaire

Qualeffo-41, the questionnaire of the International Osteoporosis Foundation, was used to assess quality of life. This questionaire is intended for use in clinical trials. The questionaire was validated in a multicentre study in seven countries involving patients with stable osteoporosis and control subjects. The domains it covers are pain, physical function, social function, general health perception, and mental function (mood) [19,20]. Total scores and individual scores of each domain were standardized to a percentage using the following formulae:

(1) $$Total\;score=\frac{\left(actual\;score-lowest\;possible\;score\right)\times 100}{score\;range}$$

(2) $$Domain\;score=\frac{\left(average\;score-lowest\;possible\;score\right)\times 100}{score\;range}$$

Domain scores are calculated by averaging the answers of one domain and transforming the scores to a score from 0 to 100. The total score is calculated by summing all answers of questions 1–41. The raw total score ranges from 41 to 205 and this is transformed to scores from 0 to 100. All answers are standardized so that 1 represents the best and 5 (or 3, or 4) represents the worst quality of life.

All patients completed the questionnaire before the start of intervention, after 3 months of exercise, and at the 3-month follow-up investigation after cessation of the exercise program.

### Ethics committee approval

Informed written consent was obtained from all patients. The study was approved by the regional ethics committee for medical research.

### Statistical analysis

All data were stored and analyzed using the SPSS statistical package 19.0 (SPSS Inc. Chicago, Illinois). Descriptive statistics were computed for variables of interest. The computed statistics included means and standard deviations of continuous variables, and are presented as mean ± SD, frequencies, and relative frequencies of categorical factors.

Testing for differences in continuous variables between the two study groups was achieved by the two-sample *t*-test for independent samples or the Mann–Whitney *U*-test by ranks as appropriate. Selection of the test was based on evaluation of variables for normal distribution, employing the Kolmogorov-Smirnov test. Fisher's exact test was used for between-group comparisons of categorical variables.

Comparisons within groups between the time points of evaluation were performed with regard to percentage changes versus baseline by one-sample *t*-tests against 0, and for percentage change between the time points named “follow-up” and “after training” by a paired *t*-test. Adjustments of alpha levels were carried out using the Bonferroni correction, i.e. the level of significance was lowered to 0.05/3 = 0.017.

All p-values resulted from two-sided statistical tests; the level of significance was set to p≤0.05 when no Bonferroni correction was required.

## Results

### Patients

**Figure 2:**
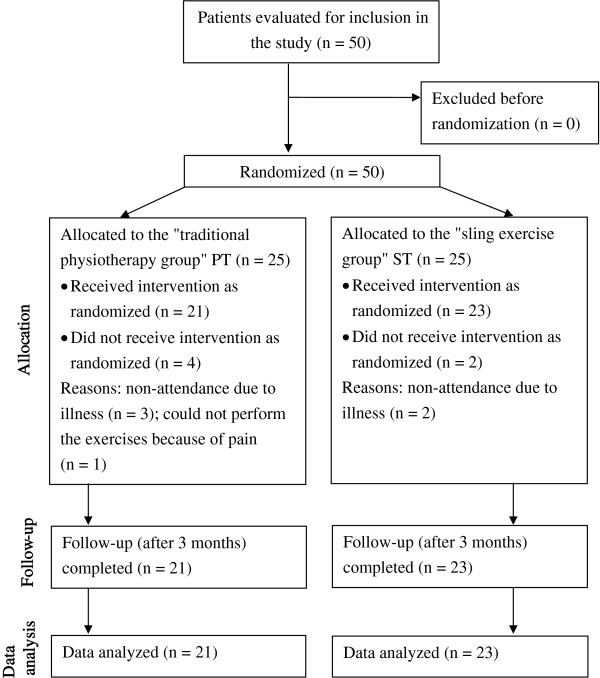
**Flow diagram for inclusion/exclusion of patients in the randomized clinical study (eligibility, allocation, follow-up, data analysis) as specified in the Consort Statement [**[21]**].**

Fifty patients with osteoporosis were initially included in the study. Of these, 25 were assigned to the PT group (conventional physiotherapy) and 25 to the ST group (sling exercises). Forty-four patients (88%) aged 62 to 84 years (mean age, 70.4 years) were followed from the start to the end of the study. Four patients in the PT group and 2 in the ST group terminated their participation prematurely. The reasons were illness or pain in the musculoskeletal system during the exercise sessions. The flow diagram in Figure 2 provides an overview of the inclusion/exclusion procedure. It was designed on the basis of the CONSORT statement (Consolidated Standards of Reporting Trials) and utilizes the common standard of randomized and controlled studies [21]. The groups did not vary significantly in respect of age (PT group 69.7 ± 3.7, ST group 71.0 ± 6.1, p = 0.409). Neither fracture rates (PT group 47.6%, ST group 65.2%) nor gender distribution varied significantly. Results for the individual domains are given below. Table 1 shows the baseline characteristics for all 44 patients.

**Table 1:** **Patient characteristics: gender: male (m)/female (f), age in years, body mass index in kg/m**^**2**^**; bone mineral density in T-score; vertebral body fractures in numbers (n); physiotherapy (PT); sling exercise therapy (ST)**

	**PT (n = 21)**	**ST (n = 23)**	**p value**
Gender m/f	2/19	2/21	1.000^Φ^
Age	69.7 ± 3.7	71.0 ± 6.1	0.409^†^
Body mass index	23.9 ± 2.9	25.6 ± 3.5	0.084^†^
Bone mineral density	−2.8 ± 0.83	−2.8 ± 0.77	0.809^†^
Vertebral body fractures	10	15	0.361^Φ^

Data are expressed as mean ± SD.

Φ Fisher's exact test, † *t*-test for independent samples between groups.

### Baseline characteristics

After three months of exercise, significant differences were registered between the PT and ST groups. These were particularly evident on pre-post comparison in the sling training group, and are interpreted as individual responses to the intervention.

### Global score

Global scores as well as scores for the domains were standardized to a 100-point scale: a score of 0 indicates no difficulties due to osteoporosis while a score of 100 indicates maximum difficulties.

The difficulties experienced by the patients were generally low (baseline: 26.0 ± 11.2 in the PT group and 29.7 ± 9.8 in the ST group; p = 0.256). The total score was significantly improved after the intervention in the ST group (29.7 ± 9.8 baseline and 24.5 ± 7.7 after the program; p = 0.002). In contrast, the overall figure for the PT group changed just slightly (26.0 ± 11.2 baseline to 25.7 ± 10.6 after training; p = 0.477). Comparison of baseline and follow-up data showed improvement in both exercise groups (26.0 ± 11.2 and 23.9 ± 10.0 in the PT group, p = 0.766; 29.7 ± 9.8 and 21.8 ± 8.1 in the ST group, p < 0.001). The change in the ST group was highly significant.

### Pain

The pain domain in the questionnaire primarily addresses back pain, which is commonly associated with osteoporosis. Patients in both groups experienced moderate pain (46.2 ± 19.3 in the PT group, 50.2 ± 23.3 in the ST group, p = 0.538). Pain levels were reduced after the intervention, and this was confirmed on follow-up as well (Table 2). The improvement in the sling exercise group was very significant (50.2 ± 23.3 baseline and 35.7 ± 22.0 after training; p < 0.001), and was even more apparent in the follow-up data (50.2 ± 23.3 baseline, 29.6 ± 24.0 on follow-up; p < 0.001). Obvious but non-significant (after Bonferroni correction) differences were observed when initial data were compared with follow-up data in the PT group (46.2 ± 19.3 baseline, 38.8 ± 23.3 at follow-up; p = 0.0278).

**Table 2:** Test results: global score, pain, physical function, social function, health perception, mental function; significance between groups; physiotherapy (PT); sling exercise therapy (ST)

		**PT (n = 21)**	**ST (n = 23)**	**p value**
Global score	Baseline	26.0 ± 11.2	29.7 ± 9.8	0.256^†^
	After training	25.7 ± 10.6	24.5 ± 7.7	0.678^†^
	Follow-up	23.9 ± 10,0	21.8 ± 8.1	0.465^†^
Pain	Baseline	46.2 ± 19.3	50.2 ± 23.3	0.538^†^
	After training	41.2 ± 23.6	35.7 ± 22.0	0.425^†^
	Follow-up	38.8 ± 23.3	29.6 ± 24.0	0.203^†^
Physical function	Baseline	16.2 ± 10.2	20.1 ± 10.4	0.210^†^
	After training	14.0 ± 9.7	13.8 ± 8.1	0.943^†^
	Follow-up	12.8 ± 8.4	10.9 ± 6.2	0.382^†^
Social function	Baseline	18.4 ± 15.2	20.3 ± 11.3	0.625^†^
	After training	16.3 ± 12.8	16.0 ± 10.1	0.924^†^
	Follow-up	17.7 ± 12.9	17.1 ± 10.4	0.864^†^
Health perception	Baseline	50.0 ± 19.0	57.6 ± 15.5	0.151^†^
	After training	49.2 ± 21.1	47.5 ± 11.6	0.743^†^
	Follow-up	44.4 ± 15.2	49.3 ± 17.4	0.334^†^
Mental function	Baseline	31.5 ± 11.6	34,3 ± 12.0	0.434^†^
	After training	38.8 ± 9.3	37.7 ± 7.7	0.678^†^
	Follow-up	34.4 ± 10.3	32.9 ± 11.6	0.645^†^

Data are expressed as means ± SD. Scale 0 (no perceived difficulty) to 100 (maximum perceived difficulty). † t-test for independent samples between groups.

### Physical function

The physical function domain includes activities of daily living, household chores, and mobility in general. Neither group reported major difficulties before the start of the program (16.2 ± 10.2 in the PT group, 20.1 ± 10.4 in the ST group; p = 0.210). The PT group experienced improvement after the intervention, which was confirmed on follow-up as well (see Tables 2 and 3). However, the changes in the PT group were not significant. The ST group, on the other hand, revealed a significant difference between scores before and after the program (20.1 ± 10.4 baseline, 13.8 ± 8.1 after the program; p = 0.001). Comparison of scores pre-program and follow-up scores revealed a highly significant improvement in the sling exercise group (20.1 ± 10.4 baseline and 10.9 ± 6.2 on follow-up; p < 0.001).

**Table 3:** Test results of percentage changes: global score, pain, physical function, social function, health perception, mental function; significance within each group; physiotherapy (PT); sling exercise therapy (ST)

	**After training vs. Baseline**	**Follow-up vs. Baseline**	**p-value**^**†**^	**p-value**^**Φ**^	**p-value**^**Θ**^
Global score					
PT (n = 21)	4.7 ± 29.9	−2.1 ± 32.3	0.477	0.766	0.179
ST (n = 23)	−14.9 ± 19.9	−25.4 ± 18.4	0.002	<0.001	0.016
Pain					
PT (n = 21)	−8.0 ± 45,6	−15.8 ± 46.6	0.442	0.147	0.278
ST (n = 23)	−28.8 ± 29.7	−47.1 ± 36.7	<0.001	<0.001	0.026
Physical function					
PT (n = 21)	−9.8 ± 42.2	−14.1 ± 41.3	0.310	0.144	0.570
ST (n = 23)	−27.2 ± 34.9	−44.0 ± 31.5	0.001	<0.001	0.014
Social function					
PT (n = 21)	−7.0 ± 44.9	18.8 ± 109.7	0.527	0.490	0.217
ST (n = 23)	8.0 ± 110.1	32.0 ± 182.3	0.731	0.409	0.339
Health perception					
PT (n = 21)	−1.6 ± 25.3	1.3 ± 55.6	0.773	0.916	0.821
ST (n = 23)	−16.1 ± 19.7	−12.5 ± 26.7	0.001	0.034	0.491
Mental function					
PT (n = 21)	34.9 ± 41.4	15.4 ± 35.5	0.001	0.060	0.075
ST (n = 23)	19.8 ± 36.5	3.4 ± 43.7	0.017	0.716	0.063

Data are expressed as means Â± SD. † comparison after training vs. baseline (one-sample t-test against 0), Φ comparison follow-up vs. baseline (one-sample t test against 0), Θ comparison follow-up vs. after training (paired t-test).

### Social function

The items of the social function domain included hobbies and activities that involved interaction with others. The data reveal little difficulties in both groups before the program (18.4 ± 15.2 in the PT group, 20.3 ± 11.3 in the ST group; p = 0.625). A marginal change was observed after the intervention (16.3 ± 12.8 in the PT group and 16.0 ± 10.1 in the ST group; p = 0.527). Similar data were recorded at the follow-up investigation (17.7 ± 12.9 in the PT group and 17.1 ± 10.4 in the ST group; p = 0.864).

### Health perception

Health perception is based on the patients' comprehensive personal assessment of their own quality of life. Patients relate their current state of health to their age, or look back on their quality of life and evaluate it. The groups were quite similar as regards their perception of health before the start of the program (50.0 ± 19.0 in the PT group, 57.6 ± 15.5 in the ST group; p = 0.151). The groups reported moderate difficulties. An improvement was observed in both groups after the intervention, but was only significant in the ST group (57.6 ± 15.5, 47.5 ± 11.6; p = 0.001). The PT group perceived their health as being improved at the follow-up investigation compared to immediately after the intervention (see Table 2). Comparing baseline and follow-up data in the PT group revealed no significant differences (50.0 ± 19.0, 44.4 ± 15.2; p = 0.916). Notable but non-significant (after Bonferroni correction) effects were registered in the sling exercise group immediately after the intervention, but were diminished thereafter (57.6 ± 15.5 baseline, 49.3 ± 17.4 on follow-up; p = 0.034).

### Mental function

This domain of the questionnaire is focused on moods and emotional states of osteoporosis patients. The difficulty experienced by patients of both groups was similar before the start of the intervention (31.5 ± 11.6 in the PT group, 34.3 ± 12.0 in the ST group; p = 0.434). Moods were deteriorated by several points in both groups (38.8 ± 9.3 in the PT group, 37.7 ± 7.7 in the ST group; p = 0.678), but initial values were nearly restored at follow-up (34.4 ± 10.3 in the PT group, 32.9 ± 11.6 in the ST group; p = 0.645). Comparisons within groups revealed a very significant difference in the PT group at the end of the intervention (31.5 ± 11.6 before the program to 38.8 ± 9.3 at the end of the intervention; p = 0.001).

## Discussion

Given a society in which the proportion of the elderly is on the increase, physicians and physiotherapists are focussing an increasing share of their attention on back pain and osteoporosis. Various exercises programs are available for patients with osteoporosis. Their efficacy as regards improving of bone density and reducing the risk of falls has been proven [8,13]. Conclusions drawn by the Cochrane Collaboration refer - among other aspects - to the improvement of vertebral bone density in postmenopausal women by means of aerobic exercises, weight-bearing exercises, and resistance exercises [8]. Our data concerning sling exercises for osteoporosis patients revealed a significant reduction in the perception of pain, a significant increase in physical performance, and a significant sensation of "feeling more healthy" among our patients. Especially worthy of mention is the enduring effect of this type of exercise on "feeling healthy" and pain relief. The effect persisted during the 3-month control period after completion of the training program. We have found no studies in the published literature reporting the results of sling exercises in patients with osteoporosis. The so-called conventional physiotherapy regimen used in our second group failed to achieve similar effects.

The relatively mild symptoms (50) experienced by our patients despite having undergone fractures is not easily explained (Table 3). Bianchi et al. [22] investigated 100 postmenopausal patients with osteoporosis, aged 50 to 85 years, in respect of quality of life and depression. The results of the study showed that osteoporosis exerted a negative impact on the psyche and quality of life of the patients. The group that experienced fractures fared the worst. One explanation for the results achieved in our study might have been the fact that 88% of our patients performed regular sports twice a week. Furthermore, our patients went for a walk at least once daily. Accordingly, the initial score for the domain *physical function* was rather low - which is indicative of a high degree of mobility in activities of daily living and household chores. This is contrasted by the relatively high level of pain before the start of the intervention. This was not significantly different in the sling exercises group (Table 3). Thus, patients benefited most in the domains of *pain und physical function*. The effect of training in this domain was perceptible even three months after completion of the program. One explanation could be the plasticity of the human neuromuscular system, which is forced to adapt consistently to new environmental conditions. In our case the unstable slings fulfill this "new" requirement. The degree of elasticity is such that the person doing the exercises is exposed to a number of stimuli. In other words, he/she has a very small supportive surface and has to regulate and control motions very rapidly and with full concentration. The deep muscles of the spine that lie close to the joints (musculus multifidus, musculus transversus abdominis, pelvic floor muscles and the diaphragm) need to be activated. This is very significant for stabilization of the lumbar spine and pain relief in this region [17,23-25]. Local stabilizers appear to be atrophied in the presence of pain, and cannot be regulated or controlled properly [25-29]. This causes instability in the respective segments of the spine. In the absence of exercise stimuli, the deep muscles are only regulated in a phasic manner. As a result an individual may lose his/her sensitivity to correct posture [26,27]. This explanatory approach has been established in the treatment of patients with back pain and the rehabilitation of sportsmen. Obviously, this fact cannot be immediately transferred to the treatment of patients with osteoporosis. However, the results of a study performed by Hongo et al. [30] show that even exercises of mild intensity may improve the strength of spinal muscles and thus exert a positive effect on quality of life in patients with osteoporosis. Circuit training as performed in Bergland et al.'s [9] study proved to be much more intensive. The authors had women with osteoporosis aged 60 to 84 years perform the training program for three months, twice a week, one hour per session, under the supervision of a physiotherapist. Based on their data Bergland et al. conclude that circuit training may exert a positive impact on mobility and quality of life. In other words, the regularity of exercise is a major factor. In order to design intensive exercises and provide individual care for patients with osteoporosis, it appears meaningful to limit the size of the group to a maximum of five persons. Immediate feedback from physiotherapists ensured that the patients were motivated, that they performed the exercises correctly (i.e. with no error), and this also increased the intensity of training. In sling exercises, the diversity of experience obtained in performing motions with the sling appears to exert a sustained effect on the sensomotor system of osteoporosis patients. Activities of daily living become easier when patients experience less pain. Improvement of sensomotor abilities appears to exert a positive impact on quality of life. Schwesig et al. [14] performed sensomotor exercises with the Aero-Step in 20 healthy elderly persons and 27 patients with osteoporosis aged on average 66.6 years. After conclusion of the training program the authors observed improvements in sensomotor performance and general wellbeing. How the authors registered the gain in quality of life is not clearly discernible from this report.

It is interesting that the domain of *Mental function* deteriorated in both groups after the intervention. We suspect that the loss of ‘new’ social contact plays a significant role.

Our study demonstrated a positive effect of sling exercises on pain, physical fitness and general body perception. Thus, sling exercises appear to be a favorable alternative to conventional physiotherapeutic procedures.

## Conclusion

It was possible with the physical therapy to improve the pain status and the functional capability of the osteoporosis patients, with the sling exercise patients benefiting most.

The combination of pain mitigation and functional improvement resulted in improved perception of health and this in turn improved the sling exercise group’s quality of life.

## Competing interest

The authors declare that they have no competing interests.

## Authors’ contributions

GS and HCS designed the study and actually wrote the article. HCS and AK contributed to select the patients with osteoporosis.GS prepared the database and made the psychological interviews. GK evaluated and calculated the test scores and performed the statistical analysis. All authors have read and approved the final version of this paper.
